# Annotations

**Published:** 1891-12-05

**Authors:** 


### Annotations.

**The Bacillus of Typhoid Fever.**

Bacteriologists seem to have almost made up their minds that they have definitely discovered the bacillus of typhoid fever. Professor Burdon Sanderson, in his Croonian Lectures, gives Dr. Koch credit for having been the first to demonstrate the character, life history, and deadly activity of this bacillus. There are certain facts about it which are practically important to all classes of readers; and, indeed, every department of bacteriology is a most fascinating study to practical physicians, and to all sorts of persons who are endowed with the scientific temper. The typhoid bacillus can be easily examined after having been photographed. It appears to be a lively little organism which throws out flagelliform processes, or long ciliary appendages, from either side of its body, and by means of these it can move about with considerable freedom. The bacillus is found not only in the particular intestinal glands which are affected in a typhoid fever case, but also in large numbers in the spleen. It is pretty evident from this latter fact that the organism infests the blood. We pointed out last week that certain bacilli thrive best when deprived of air, whilst certain others flourish most luxuriously in abundance of air or oxygen. The bacillus of typhoid can live and thrive either with or without air. It prospers, as we know, in the intestinal glands, and it can equally find a suitable nidus on an external cultivation medium; that is to say it is both aërophytic, and anaërophytic. Dr. Burdon Sanderson informs us of two very important practical facts, namely, that the typhoid bacillus grows luxuriantly in milk, and is also capable of vegetating in drinking water. It is plain, then, that whoever habitually drinks uncooked milk or even unboiled water, may at any moment, without perceiving any peculiarity at all in either the water or the milk he is drinking, gulp down whole armies of active typhoid bacilli, and thus acquire typhoid fever, he knows not how or where. Undoubtedly in England, where cows are kept under all sorts of insanitary conditions, and where even the purest water is liable to be contaminated by unsuspected sewage, it should be a standing rule in every household to boil both water and milk before using. There is one gleam of comfort, perhaps there are two, in Dr. Burdon Sanderson's latest conclusions. He says that it has not yet been experimentally proved that typhoid is really caused by what is called Eberth's bacillus; and further, that it is very likely we shall never succeed in communicating typhoid fever to animals. This is so far encouraging. We may still eat beef and mutton, even if it be a little underdone, without fear that this particular fever will result. But the probability that Eberth's bacillus does give rise to typhoid is, after all, so very great that all of us ought unhesitatingly and for ever to declare against the use of unboiled water and milk.

**Leech's Life.**

It will be news to many that John Leech was designed for a doctor. Yet so it was. He studied physic at St. Bartholomew's with Albert Smith, Percival Leigh, and Gilbert à Becket. It is almost a pity that he did not enter the profession, and illustrate its humours with his comic pencil. Many of the Fillgraves of our order might have enjoyed a posthumous immortality which has been denied them if he had done so. Mr. Frith, R.A., has acted Boswell to Leech's Johnson; and has produced a book which has at least one of the merits of Boswell's famous biography, in that it is readable to the last page. Among other things we are shown a picture of the interior of a London general practitioner's sanctum of forty years ago, which is both edifying and amusing. Leech was apprenticed to one Whittle. That gentleman, it seems, was a pigeon fancier by preference, and only a doctor by necessity. He was somewhat of a German in his capacity for beer and tobacco. It is not unlikely that in that respect he was the type of a large class. Probably, there may be found here and there a few survivals of similar "unfittest" at the present day. Whittle's house was a menagerie with a dispensary in it. Every spare inch of ground was occupied by rabbits, ferrets, guinea-pigs, and an infinite variety of fowls. This genial professional lived by doctoring the police and selling cheap nostrums to the poor. One drawer in his medicine-chest contained pills so constructed as not to do any harm. These were for the consumption of the poor, and were composed of bread and soap. The drawer bore the appropriate label of "Pil-Hum," which was short for Pilula Humbugana. From Whittle, Leech went to Cockle, who is said to have been a son of the inventor of another notorious pill. Leech did a great deal for the English of the middle and lower middle class. Who knows how much of its present decency and simplicity of behaviour it owes to him? If he had but remained a doctor, and issued an occasional medical *Punch*, we might all have been less pedantic, and more human than we are at the present time. A little wholesome ridicule has its value even for the most perfect of mortals. We do not say that doctors as a class are perfect in the superlative degree; we are only anxious, as every loyal member of the profession should be, for them to become so; and we would willingly welcome the help of a Leech or a Du Maurier in a task which is by no means the easiest in the world.

**Direct Representation: "Who Killed Cock Robin?"**

It is most decidedly an amusing world. There is far more effective difference in rank between the village postmaster and the village postman than there is between the Prime Minister and the House of Lords' policeman. The members of the General Medical Council in their corporate capacity sometimes illustrate this funny divergence of rank in quite remarkable ways. At the last meeting of this body, Sir Walter Foster brought forward a motion to the effect "that in the opinion of the Council, the time has come when it would be expedient to confer upon the registered medical practitioners, resident in the United Kingdom, the power of returning an increased number of direct representatives to the General Medical Council as provided for in section 10, sub-section 1, paragraph C of the Medical Act (1886)." Sir Walter supported his resolution by the general contention that the profession wished to appoint at least one more direct representative to the Council, and that it would not only give satisfaction to the great body of practitioners, but would improve the efficiency of the Council if his motion were carried into effect. There was nothing very alarming about the proposal, and as certainly there was nothing unreasonable. As a matter of fact the composition of the Council would remain practically unaltered if the change were made, and there is, therefore, no reason whatever, why a body of fairly educated men, such as the members of the medical profession are, should not have their reasonable wishes gratified. But how did the non-elected members, the life peers so to speak, of the Council, receive the proposal? Sir William Turner suggested that the prospect of an election had given wings to Sir Walter Foster's eloquence. That, at least, was his utterance, minus the figure of speech. Sir William is not given to metaphor. What, he asked, had the Medical Council to do with the hardships and struggles of professional life? Nothing, certainly! Such distinguished eminences as professors of anatomy, who, thirty or forty years ago, probably knew what it was to occasionally have a difficulty in paying their landlady's modest demand for rent on a Saturday night, can have no sympathy with the hardships and struggles of professional life. Of course not! The profession knows well that teachers of anatomy are not only now, but always have been eminent persons, and do not know the obverse of a half-crown from the reverse. Sir William Turner is notoriously indifferent to the value of money. Every first and second year's student of Edinburgh University knows that perfectly well. Sir Dyce Duckworth thought the demand for direct representation altogether unnecessary. Quite so. Cynical people, after reading Sir Dyce's speech, might venture upon the opinion that it was "altogether unnecessary." Sir John Simon held that Sir Walter Foster's proposal was an essentially frivolous motion. Sir John is an "essentially" funny man. Of course, it was a frivolous motion; anybody ought to have known that. "Who killed Cock Robin? I, said the sparrow." Sir John Simon plays the part of the sparrow to perfection; and he has won for himself the enduring distinction of having at least tried to kill Cock Robin. Mr. B. Carter expressed a fear lest the future of direct representation might fall into the hands of "carpet baggers." Dreadful people those "carpet beggars"! Shocking people! We deeply sympathise with Mr. Carter's horror of them. But what exactly is a "carpet bagger" in the medical profession? We are but simple people and would like to know. Perhaps Mr. Carter will tell us. Truly, the whole performance was a most amusing and edifying farce. One does not need to go outside the Medical Council to know where Anthony Trollope got his doctors.

